# Nanocrystals as a promising approach for enhancing solubility and dissolution of etoricoxib using Box–Behnken design

**DOI:** 10.1038/s41598-025-12837-3

**Published:** 2025-08-11

**Authors:** Lamiaa M. Ahmed, Fergany A. Mohamed, Tahani H. Elfaham

**Affiliations:** Department of pharmaceutics, Faculty of pharmacy, Assiut University, Assiut, 71526 Egypt

**Keywords:** Etoricoxib, Nanocrystals, Box–Behnken, Saturation solubility, Dissolution, Drug discovery, Nanoscience and technology

## Abstract

Poor solubility of drugs represents a major obstacle against drug delivery, so pharmaceutical industry is exploring the use of nanocrystals as a promising approach to enhance the bioavailability of those medications with improving their pharmacokinetics. This study aims to improve etoricoxib properties via nanocrystal form using an acid-base precipitation method. This method is simple, environment- friendly, and prefers non-organic solvents and chemicals, thus overcoming challenges in developing dosage forms. Prepared nanocrystals were optimized for several factors such as stabilizer type and concentration, amount of drug, time and speed of homogenization. FT-IR, DSC, X-ray diffraction, and TEM characterizations were conducted on the optimized nanocrystal formula. The findings showed a successful inclusion of etoricoxib as nanocrystals with a mean particle size of 210.30 ± 10.20 nm, PDI of 0.277 ± 0.01, and a zeta potential of − 74.10 ± 0.61 mV. TEM imaging revealed well-defined cubic-shaped nanoparticles, indicating morphological uniformity and excipient compatibility. Solubility studies demonstrated notable enhancement in the aqueous solubility of etoricoxib nanocrystals (137.75 ± 1.34 µg/mL) compared to the pure drug (87.70 ± 1.41 µg/mL). Additionally, the nanocrystals exhibited rapid dissolution profile, achieving 91.49 ± 0.01% drug release within 5 min. These results suggest that using nanocrystals to improve the aqueous solubility and dissolution of medications with poor solubility is a potential strategy.

## Introduction

Solubility is a key factor in the effectiveness of curative dosage forms^1^. It significantly influences a drug’s oral absorption, bioavailability, and overall pharmacokinetic behavior. Generally, higher solubility leads to better dissolution and enhanced absorption^2^. According to the Biopharmaceutical Classification System (BCS), Class II drugs are characterized by poor aqueous solubility, which presents significant challenges in terms of oral absorption and bioavailability^3^. Various methods had been taken into consideration to improve the solubilization and dissolution of those medications, including micronization, solid dispersion and complexation^4^. Drug micronization; a size reduction in which fewer than 10 microns of particle size is produced, but it is not recommended since the product’s tendency to clump together reduces its effective surface area for dissolving^5,6^. Solid dispersion technique, despite its positive impact in improving drug solubility and dissolution, is very limited for commercial use due to several drawbacks such as costly and time-consuming preparation methods, challenges integrating it into dosage formulation, and problems with scaling up the manufacturing process^7,8^. As for complexation; the creation of complexes between the medication and a ligand via Intermolecular associations (covalent or non-covalent bonds), yet this method has drawbacks, e.g. the chemical must be able to form complexes with ligands^9^. For compounds with very limited solubility, solubility enhancement may be quite modest. This also applies to solubilization by combination processes, such as complexation with pH adjustment. Lastly, the presence of a ligand can lead to problems with quality control, regulations, and potential toxicity, which can make the development process more difficult and costly^10^. Another crucial method is nanosization, which improves drug solubility by increasing surface area and particle wettability via particle size reduction into nanoscale^11^. The process of nanosization involves creating drug nanoparticles (nanocrystals) or nano-carriers (lipid and polymeric nano-systems)^12,13^. Nanocrystals (NCs), pure active pharmaceutical ingredients (APIs) with a size range of 10–1000 nm, have gained attention in pharmaceutical innovation in recent decades, especially for improving the bioavailability of drugs that are poorly soluble^14^. NCs are simple and economical formulating procedures that primarily rely on the active ingredients themselves with the subtle addition of small amounts of polymers or surfactants for stabilization properties; adsorbing at the NPs’ surface and preventing the drug particles from aggregating, so NCs overcome problems associated with the counteracting other approaches^15,16^. Compared to other nano-systems, nanocrystals offer several benefits, such as the ability to achieve maximum drug loading. As a result, a lower dosage is taken into consideration, improving bioavailability and therapeutic efficacy with lowering adverse effects. Also, NCs require a low quantity of surfactants for stabilization so reduce the related toxicity and provide more safety profiles^15,17^. NCs can be prepared using various methods, including top-down techniques such as high-pressure homogenization and milling. In these approaches, the starting material consists of larger solid particles, which are reduced to the nanoscale through mechanical force. A key advantage of these methods is the avoidance of organic solvents^18^. However, high-pressure homogenization has certain limitations: it requires thermostable components, involves high process temperatures and energy input, demands complex equipment, and may lead to thermal or mechanical degradation of the drug, potentially resulting in reduced yield^19^. Similarly, the milling technique is associated with drawbacks such as metal contamination from the milling media, prolonged processing times, high energy consumption, and reduced crystallinity due to mechanical stress^20^. In contrast, the acid–base precipitation method relies on the pH-dependent solubility of hydrophobic drugs, where the drug is first dissolved in an acidic or basic medium and then precipitated by neutralization. This approach is simple, cost-effective, rapid, and does not require complex equipment or the use of organic solvents, making it a more environmentally friendly alternative. Given these advantages, the acid–base precipitation method was selected in this study for NC preparation, with a focus on critical formulation and process optimization^21^. Etoricoxib (ETX), 5-chloro- 6′-methyl- 3[4-(methyl sulfonyl) phenyl] − 2, 3′–bypyridine, is a highly potent and selective cyclooxygenage-2 (COX-II) inhibitor compared to celecoxib and rofecoxib, ETX is administered orally as an analgesic and nonsteroidal anti-inflammatory drug^22,23^. Compared to COX-1 inhibitors, ETX has substantially fewer upper gastrointestinal issues and is used to treat rheumatoid arthritis, osteoarthritis, postoperative tooth pain, chronic back pain, and acute gout^24^. It is categorized as a low-solubility chemical since ETX is a class II, weakly basic compound (pKa of 4.6) with reduced solubility in the pH 4–7 range^25^. Improving the solubility of ETX helps to improve its dissolving behavior, which in turn improves its pharmacokinetics and therapeutic efficacy^26^. Numerous studies have attempted to improve ETX solubility through various techniques, which subsequently increased its bioavailability^27–29^. This study aimed to investigate the efficacy of nanocrystals made using the safest and simplest method in enhancing the dissolution behavior of etoricoxib.

## Materials and methodology

### Materials

Etoricoxib (EX) was a kind gift from APEX PHARMA, Cairo, Egypt, poloxamer407 (F127) and mannitol were purchased from sigma-Aldrish. St. Louis, MO, USA and LANXESS Co., Cologne, Germany respectively, soyabean lecithin was obtained from alpha chemika Co., Cairo, Egypt. All other chemicals were of analytical grade and utilized as received.

### Methodology

#### Preparation of ETX-NCs

ETX-NCs were prepared using acid-base precipitation method^30^. In this process, a specific amount of ETX was dissolved in a 0.5 M HCl solution, while a stabilizer was dissolved in a solution of NaOH with certain molar concentration, both solutions were formed under magnetic stirring. The acidic solution was then slowly added to the alkaline solution under homogenization at a controlled speed and for a set duration. Freeze drying of formed nanosuspension was conducted to form nanocrystals in powder form using mannitol (5% w/v) as a cryoprotectant.

#### Optimization of NCs

##### Screening of stabilizer type, pH of the medium and stabilizer concentration

Various stabilizers were tested while keeping other parameters constant to determine the most effective one for ensuring the stability of the prepared ETX-NCs. The evaluation was based on particle size, PDI, and zeta potential measurements. Once the most suitable stabilizer was identified, the pH of the medium and stabilizer’s concentration were adjusted accordingly. Table 1 shows the composition of ETX-NCs formulae.

**Table 1:** Preparing conditions of NCs formulae.

Formula no.	Stabilizer type	Conc. of stabilizer (%w/v)	pH	Amount of ETX (mg)	Homogenizer speed (rpm)	Homogenizer time (min)
F1	PEG 6000	0.1	8.5	30	21,000	5
F2	PVP	0.1	8.5	30	21,000	5
F3	HPMC	0.1	8.5	30	21,000	5
F4	Pluronic 68	0.1	8.5	30	21,000	5
F5	Lecithin	0.1	8.5	30	21,000	5
F6	Pluronic 127	0.1	8.5	30	21,000	5
F7	Tween 80	0.1	8.5	30	21,000	5
F8	Kolliphore EL	0.1	8.5	30	21,000	5
F9	Brij 35	0.1	8.5	30	21,000	5
F10	SLS	0.1	8.5	30	21,000	5
F11	Lecithin	0.1	7.4	30	21,000	5
F12	Lecithin	0.1	5.5	30	21,000	5
F13	Lecithin	0.025	5.5	30	21,000	5
F14	Lecithin	0.05	5.5	30	21,000	5
F15	Lecithin	0.15	5.5	30	21,000	5
F16	Lecithin	0.2	5.5	30	21,000	5
F17	Lecithin	0.4	5.5	30	21,000	5

##### Optimization of ETX-NCs using Box–Behnken design

The type and concentration of the selected stabilizers were kept constant based on the results of preliminary optimization (Table 1). To optimize other formulating conditions of NCs, a Box–Behnken design with 3 factors and 3 levels was employed, focusing on the amount of ETX (mg), homogenization speed (rpm), and homogenization time (min), using Design Expert, version13 software. The Box–Behnken design was chosen for its efficiency in saving time and cost by minimizing the number of runs required for optimization. A total of 17 runs were performed, including 5 replicated center points, where each run evaluated for particle size, PDI, and zeta potential. The independent variables considered were the amount of ETX (A), homogenization speed (B), and homogenization time (C), while the dependent variables were particle size (Y1), PDI (Y2), and zeta potential (Y3). The levels of independent and dependent variables are shown in Table 2.

**Table 2:** Box–Behnken design with independent and dependent variables for optimization of ETX-NCs.

Factors (independent variables)	Levels		
	− 1	0	+1
A: Amount of ETX (mg)	30	40	50
B: Speed of Homogenizer (rpm)	6500	13,750	21,000
C: Time of Homogenizer (min)	2	5	8

Responses (dependent variables)	Constraints		
Y1: Particle size (nm)	Minimum		
Y2: PDI	Ranged (0.252,0.290)		
Y3: Zeta potential (mV)	Maximum		

## Characterization of prepared NCs

### Particle size, PDI and zeta potential

The Malvern Zetasizer Nano Series, utilizing dynamic light scattering (DLS) technology^31^was employed to measure particle size, PDI, and zeta potential of the prepared formulations. Prior to analysis, the formulations were diluted 100-fold with distilled water to ensure accurate readings.

### Drug content

A specified weight of NCs (equivalent to 340.9 µg) was dissolved in a 10 mL volumetric flask containing a mixture of phosphate buffer (pH 7.4) and methanol in a 9:1 ratio. The NCs were initially dissolved in 1 mL of methanol, followed by vortexing for 5 min. Then, 9 mL of phosphate buffer (pH 7.4) was added under continuous vortexing. The drug content of ETX was measured using UV spectrophotometer at λ _max_ of 235 nm against blank (similarly treated) after suitable dilutions. Drug content was detected using the following equation^32^:

1 $${\text{Drug content }}\% =~\frac{{Actual~~amount~of~drug~in~NCs~}}{{Theoretical~amount~of~drug~in~NCs~}} \times 100$$

### Electron microscopy analysis

Samples (raw ETX and ETX-NCs) were examined for morphology using transmission electron microscopy and scanning electron microscopy. For TEM analysis, 5 µL of the prepared NCs suspension was placed onto a carbon-coated copper grid, allowing the NCs to settle for 3–5 min. After settling, the excess fluid was removed by blotting with absorbent paper. The grid was then negatively stained with 2% uranyl acetate for 3–5 min. Finally, digital images of the NCs were captured using a Gatan axis-mount 2k x 2k digital camera. While for SEM determination, the monolayer of each sample was mounted on a metallic stub and coated with a thin 10 nm layer of gold using the sputter coating technique. The samples were then examined under the SEM at an accelerating voltage of 15 keV^33^.

### Fourier infrared spectroscopy (FT-IR)

The infrared spectra of intact ETX, Lecithin, Mannitol, the selected ETX-NCs formulation, physical mixture of NCs components (ETX, lecithin, mannitol) and the physical mixture of ETX-NCs without mannitol (ETX, lecithin) were recorded using FT-IR spectrophotometer (Shimadzu IR-345, Japan) to investigate any potential interactions between ETX and the excipients used in the NCs formulation. Each sample was individually grounded with potassium bromide and compressed into disks. Spectral scanning was conducted in the range of 500 to 4000 cm^−1^^34^.

### Differential scanning calorimetry (DSC)

Differential Scanning Calorimetry (DSC) was employed to assess the physical state of ETX and evaluate potential drug-excipient interactions within the NCs. The DSC analyses were conducted on samples of ETX, lecithin, mannitol, ETX-NCs formula, physical mixtures of the NCs components (ETX, lecithin, and mannitol), and physical mixture without mannitol (ETX, lecithin) using a computer-interfaced Shimadzu calorimeter. Approximately 5 mg of each sample were precisely weighed, sealed in an aluminum pan, and heated at a rate of 10 °C/min across a temperature range of 25–300 °C^35^.

### Powder X-ray diffraction (PXRD)

Samples of ETX, lecithin, mannitol, a physical mixture of ETX, lecithin, and mannitol, a physical mixture of ETX and lecithin, and the optimized NCs formula were analyzed using X-ray diffractometer (Philips PW 1710) using Cu Kα radiation (tube operated at 40 kV, 100 mA). Data were collected over an angular range from 4 to 60° 2θ in continuous scan mode, with a step size of 0.06° 2θ and a time counting of 60 s per step^36^.

### Saturation solubility

To evaluate the impact of formulating ETX as NCs on its saturation solubility, samples of pure ETX, a physical mixture (ETX, lecithin, and mannitol) in the same ratios as in the NCs, and the optimized NCs preparation were added in excess to vials containing 10 mL of phosphate buffer solution (pH 7.4). The vials were shaken at 100 rpm under thermostatic conditions of 37 ± 0.5 °C for 72 h. The samples were then filtered using a 0.45 μm syringe filter, diluted, and analyzed using UV spectrophotometer at λ max of 235 nm against a similarly processed blank^37^.

### In-vitro drug dissolution

The paddle type dissolution apparatus (USP apparatus 2, Electrolab, TDT-06 N, India), was employed to study the dissolution of ETX and its nanocrystal form. Samples of pure ETX, a physical mixture (ETX, lecithin, and mannitol), physical mixture (ETX and lecithin), physical mixture of ETX and mannitol and the selected ETX-NCs, each equivalent to 8 mg of ETX, were added to 350 mL of phosphate buffer (pH 7.4) maintained at 37 ± 0.5 °C for 4 h. The paddles were set to rotate at 75 rpm. At specified time intervals (5, 10, 20, 60, 120, and 240 min), 5 mL aliquots were withdrawn and were replaced with fresh dissolution medium to maintain sink conditions. The withdrawn samples were filtered using a 0.45 μm syringe filter and analyzed by UV spectrophotometry at λ max of 235 nm against a similarly prepared blank^38^.

### Statistical analysis

Both the Newman-Keuls post-hoc test and one-way analysis of variance (ANOVA) were used to determine the significance between the various groups (* (*P* < 0.05), **(*P* < 0.01), ***(*P* < 0.001), and NS (non-significant). Each experiment was carried out in triplicate, and the mean ± SD was used to display the data.

## Results and discussion

### Preparation and optimization of ETX-NCs

**Fig. 1:**
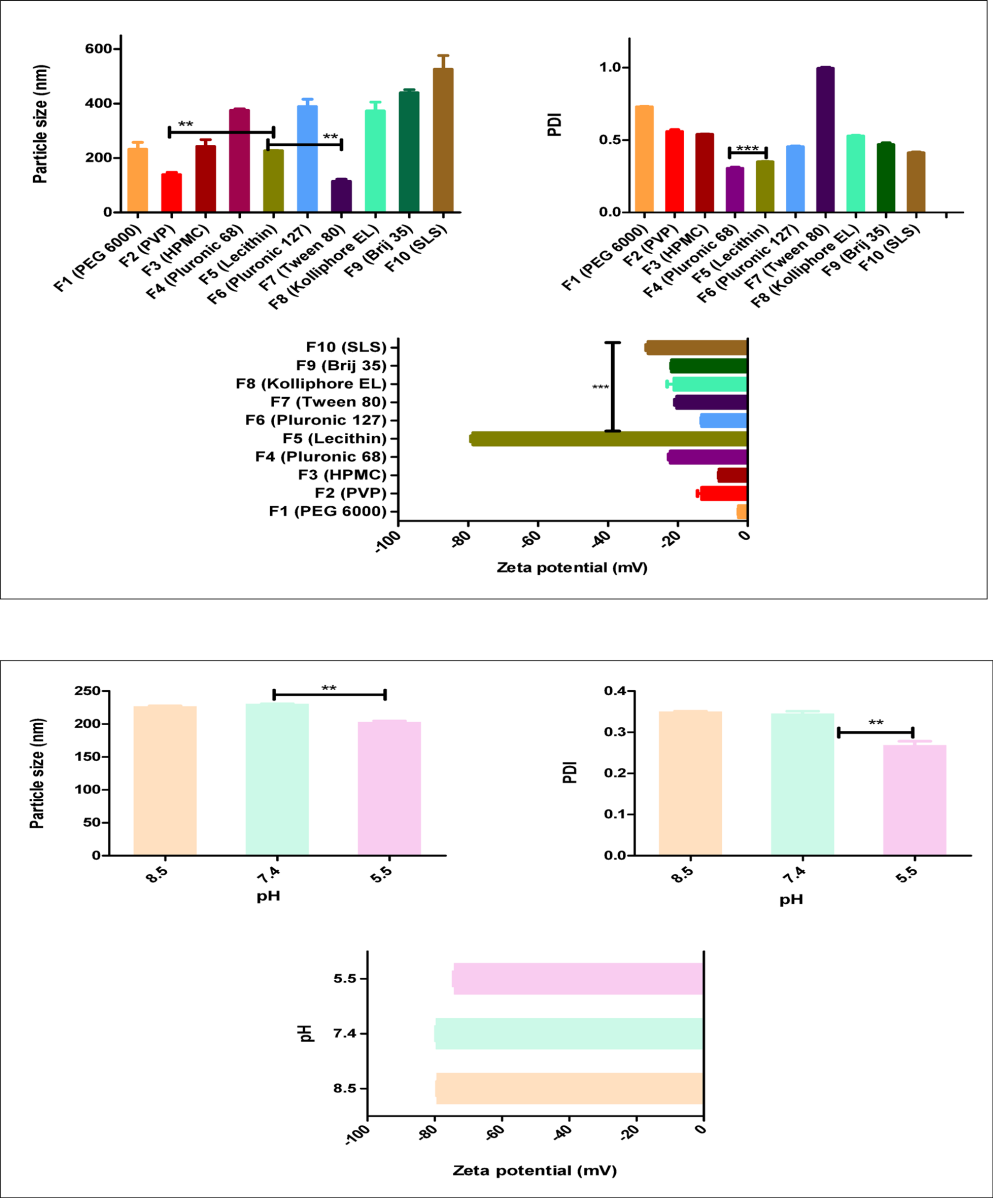

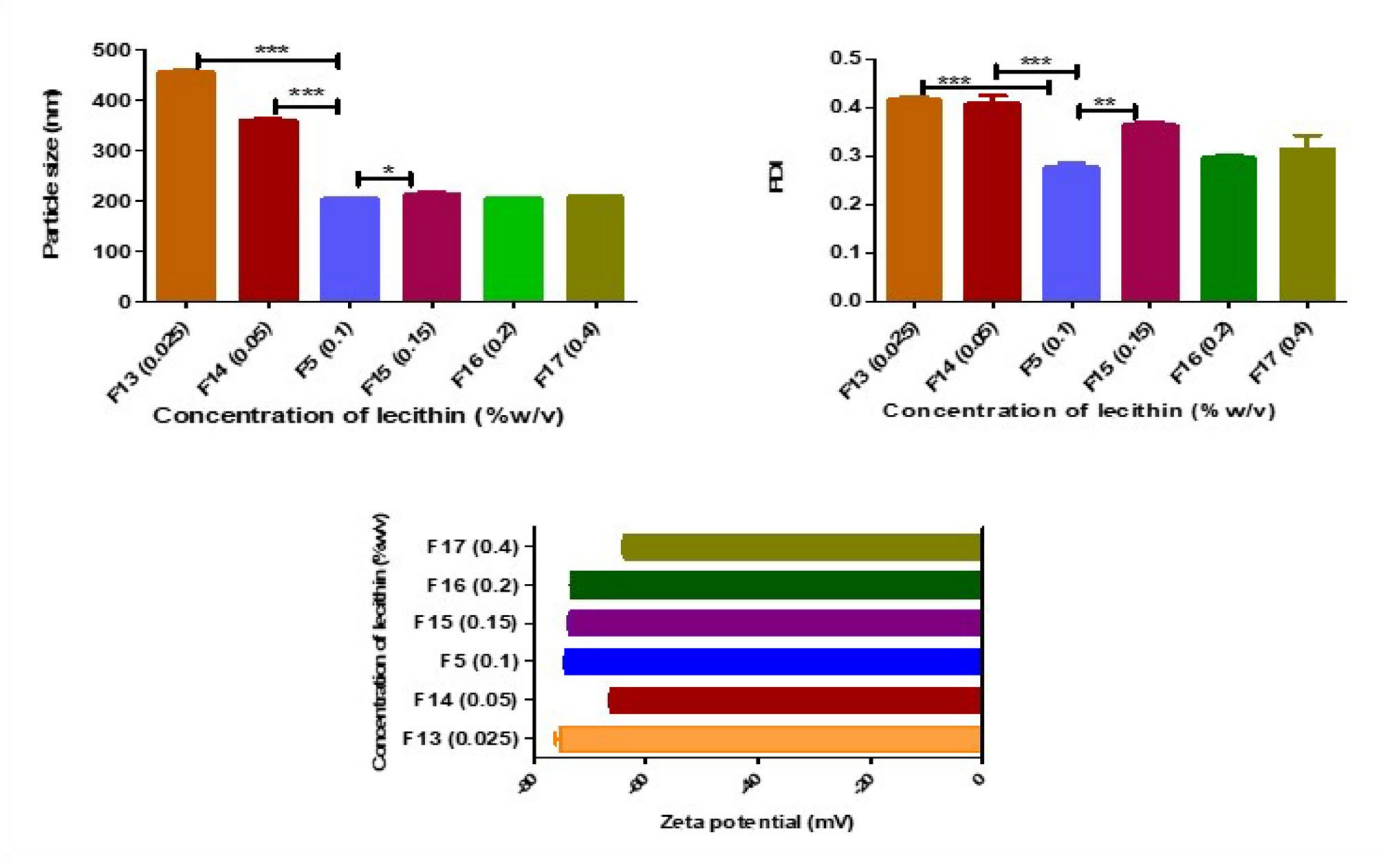
**a** Effect of different stabilizer types on particle size, PDI and zeta potential, one way ANOVA was used for statistical analysis; *** (*P* < 0.001), **(*P* < 0.01). **b** Effect of pH of the medium for NCs formulation on particle size, PDI and zeta potential, one way ANOVA was used for statistical analysis; ** (*P* < 0.01). **c** Effect of different concentrations of lecithin on particle size, PDI and zeta potential, one way ANOVA was used for statistical analysis; *** (*P* < 0.001), ** (*P* < 0.01), *(*P* < 0.05).

The acid-base precipitation method was effectively employed to prepare ETX-NCs. Investigating the stabilizing impact of several stabilizer types throughout the ETX-NCs preparation, lecithin was found to be the most appropriate one achieving NCs (F5) with tiny PS (226.65 ± 1.20 nm, p value < 0.001), low PDI (0.349 ± 0.00, p value < 0.001) and maximum ZP (-78.50 ± 0.63 mV, P value < 0.001) in comparison to other tested stabilizers (Fig. 1a). Lecithin’s substantial stabilizing impact is mostly attributed to the steric barrier that its bulky structure creates around the nanoparticles. Consequently, repulsive forces are in effect. Also, the stabilizing effect may be aided by the amphiphilic character of lecithin, which permits the hydrophobic interaction between the hydrophobic lecithin tail and the ETX hydrophobic areas^39–41^. The ETX-NCs formulated with Tween 80 and PVP exhibited a reduced size (114.50 ± 7.77 and 138.80 ± 8.06, p value < 0.01) compared to those prepared with lecithin (Fig. 1a). However, their PDI was notably higher (0.99 ± 0.01 and 0.559 ± 0.01, p value < 0.001) indicating a decrease in stability (-20.25 ± 0.64 and − 13.15 ± 1.20, p value < 0.001) respectively. While for formulations prepared with Pluronic F68, higher PS and lower zeta potential were obtained (375.25 ± 5.16 and − 22.1 ± 0.57, p value < 0.001) with minimum PDI (0.304 ± 0.01, p value < 0.001) compared to that prepared with lecithin (Fig. 1a). Considering the pH of prepared ETX-NCs, three distinct pH levels were investigated (5.5, 7.4, and 8.5) to obtain the best conditions for preparation. The results cited in Fig. 1b, illustrate the effect of lowering pH in enhancing NCs characters; NCs prepared at pH 5.5 achieved significantly lower particle size (PS) and polydispersity index (PDI) values of 204.45 ± 2.19 (*p* < 0.01) and 0.277 ± 0.00 (*p* < 0.01), respectively, compared to those prepared at higher pH levels (7.4 and 8.5). This effect can be attributed to the influence of pH changes on the nucleation and growth dynamics of the system. At pH 5.5, the system likely undergoes supersaturation more rapidly, promoting homogeneous nucleation, which leads to the formation of smaller and more uniform nanoparticles. In contrast, as the pH increases, the rate of precipitation slows, and heterogeneous nucleation becomes more prominent, resulting in larger and less uniform particles^42–44^. Furthermore, etoricoxib is a weak base (pKa ≈ 4.6) with pH-dependent solubility, showing increased solubility at alkaline pH. At higher pH values, the delayed onset of nucleation due to increased solubility allows for faster particle growth once nucleation begins, which contributes to the formation of larger particles with a broader size distribution, compared to its behavior at pH 5.5^25,45^. Considering zeta potential, it was found that raising the pH (7.4 and 8.5) achieved more negative values (-79.50 ± 0.35 and − 78.50 ± 0.63) than that observed at pH 5.5 (-74.50 ± 0.28), (Fig. 1b) which may relate to more ionization of phosphate group of lecithin at higher pH^39,40^. As a conclusion, stable ETX-NCs were obtained at the different examined pH conditions. Based on those results, pH 5.5 was selected for further evaluations. By changing lecithin concentration (0.025,0.05,0.1,0.15,0.2 and 0.4%w/v), it was found that 0.1%w/v was the optimum concentration, Fig. 1c, with small PS of 204.45 ± 2.19, low PDI of 0.277 ± 0.00 and high zeta potential of -74.50 ± 0.28. Also, it was shown that smaller concentrations of lecithin (0.025% and 0.05% w/v) resulted in larger PS and PDI (453.85 ± 3.18 nm, 0.417 ± 0.01 and 359.60 ± 1.83 nm, 0.408 ± 0.02, p value < 0.001) respectively. However higher concentrations (0.15%, 0.2% and 0.4%) resulted in slight increase in PS and PDI (212.35 ± 2.62 nm, 0.364 ± 0.01, 205.30 ± 0.71 nm, 0.296 ± 0.01 and 207.00 ± 1.41 nm, 0.314 ± 0.03), Fig. 1c. Based on these results, the best formula for further optimization was found to be F5 (0.1% w/v of lecithin, 30 mg ETX, homogenized at 21000 rpm for5 minutes, pH 5.5), since it has the lowest surfactant content, resulting in the lowest PS, monodispersity index, and highest stabilization of the generated NCs.

## Optimization of ETX-NCs by Box–Behnken design

**Fig. 2:**
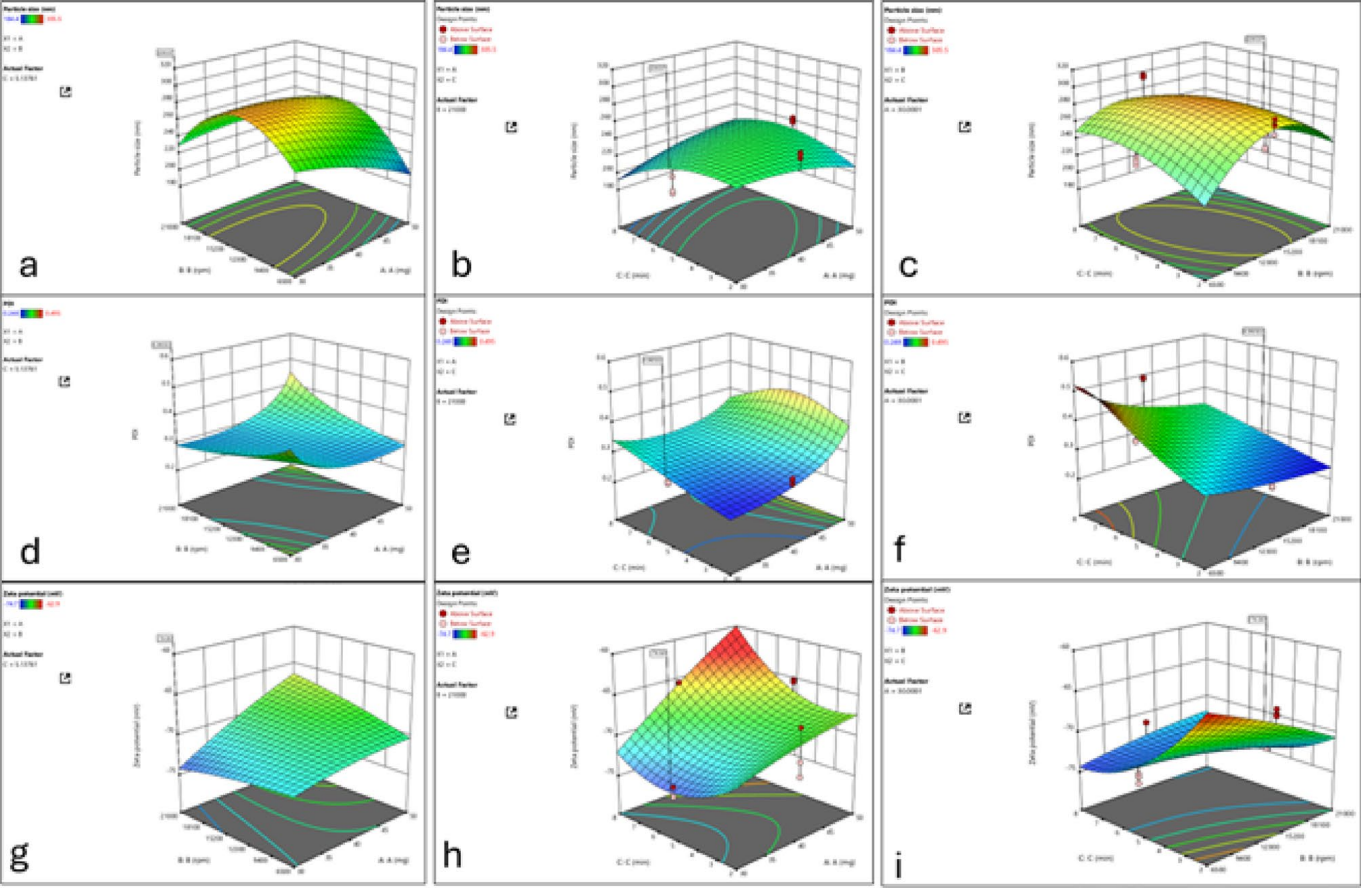
3D Response surface plots for the effect of factors, A; ETX amount (mg), B; homogenization speed (rpm), and C; homogenization time (min), on particle size (**a**–**c**), PDI (**d**–**f**) and zeta potential (**g**–**i**).

Table 3 displays the makeup and outcomes of the impact of independent variables on the responses under study. F5 was recommended by the software. Numerical optimization based on the desirability function approach resulted in an overall desirability score of 0.777, reflecting an acceptable compromise among the targeted formulation goals namely, the minimization of particle size and polydispersity range (0.252,0.290), and the maximization of zeta potential. The optimal formulation conditions yielded minimum size with low PDI and maximum zeta potential (210.30 ± 10.20 nm, 0.277 ± 0.01 and − 74.10 ± 0.61, respectively). ANOVA (analysis of variations) was used to analyze the responses and forecast the optimal parameters and mathematical model. The individual and combined factors^,^ coefficients and p-values showed how each component affected the chosen responses. The relevance of the used model and other parameters evaluated by ANOVA was also shown by the p-value; a p-value of < 0.05 denotes significance. A significant quadratic model was found for every response (Table 5). The final coded regression equation of the applied quadratic model for each individual response, including particle size (Y1), PDI (Y2) and Zeta potential (Y3), generated by response surface methodology using Design Expert^®^ software, are provided below. Figure 2 displays the 3D response surface plots of all the responses illustrating the effects of various factors.

### Model fit analysis

To find the best fit model, several tests were conducted on various polynomial models, linear, interactive (2FI), quadratic, and cubic, such as using sequential model sum squares, lack of fit test, and model summary statistics. Regarding sequential model sum squares test and model summary statistics results (Table 4), it was found that 2FI and quadratic models were significant with p values of 0.0435 and < 0.0001 for PS and p values of < 0.0001 and < 0.0001 for PDI respectively. However, zeta potential showed significance with the three models (linear; p value 0.0008; 2FI, p value < 0.0001 and quadratic model; p value < 0.0001). To detect the best fit model, F values, PRESS values, adjusted R^2^and predicted R^2^ for every response should be considered and compared for all significant models as the model that exhibits higher F value, lower PRESS value with maximum adjusted R² and Predicted R² values is regarded as the best fit model^46^. For PS and ZP responses, higher F values were observed with quadratic model (37.88 for PS and 19.56 for ZP) opposed to that in 2FI model (2.94 for PS, 13.19 for ZP) and 6.59 for linear model in ZP, Table 4. However, F value for PDI was slightly lower in quadratic model (11.16) than that for 2FI model (12.26). Regarding PRESS values, the quadratic model showed the lowest values in all responses (20863.68 for PS, 2868.29 for PDI and 127.35 for ZP) compared to others, Table 4. For adjusted R² and Predicted R² values, quadratic model presented the maximum values in all responses (0.7660 and 0.6824 for PS, 0.6673 and 0.5245 for PDI, 0.8141 and 0.7433 for ZP) respectively, compared to other significant models, Table 4. Additionally, there is less than 0.2 discrepancy between the adjusted and predicted R^2^ values, indicating a reasonable level of agreement. These findings indicate that the quadratic model fits all responses the best. Because of its aliasing, the cubic model was removed and not taken into consideration. All responses showed a significant lack of fit test for the chosen model (p value < 0.0001) with unfavorable F values of 38.49 for PS, 50.51 for PDI, and 8.00 for ZP^46^.

**Table 3:** Composition and effect of different factors on the responses studied.

Formula no.	ETX amount (mg)	Homogenizer speed (rpm)	Homogenizer time (min)	PS (nm)	PDI	ZP (mV)
F18	30	6500	5	238.4 ± 4.7	0.401 ± 0.003	− 72.8 ± 0.61
F19	50	6500	5	212.7 ± 5.7	0.305 ± 0.019	− 70.3 ± 1.76
F5	30	21,000	5	210.3 ± 10.2	0.277 ± 0.005	− 74.1 ± 0.61
F20	50	21,000	5	240.9 ± 2.5	0.415 ± 0.006	− 65.2 ± 0.17
F21	30	13,750	2	277.0 ± 8.6	0.252 ± 0.004	− 65.1 ± 0.41
F22	50	13,750	2	189.5 ± 1.0	0.294 ± 0.010	− 66.4 ± 1.15
F23	30	13,750	8	294.9 ± 1.7	0.492 ± 0.003	− 72.7 ± 1.32
F24	50	13,750	8	265.4 ± 8.0	0.310 ± 0.016	− 65.2 ± 0.70
F25	40	6500	2	186.5 ± 1.8	0.288 ± 0.017	− 63.7 ± 0.80
F26	40	21,000	2	246.1 ± 3.2	0.281 ± 0.009	− 69.7 ± 3.02
F27	40	6500	8	225.8 ± 1.8	0.259 ± 0.002	− 69.8 ± 0.11
F28	40	21,000	8	206.8 ± 1.8	0.288 ± 0.006	− 65.7 ± 0.25
F29	40	13,750	5	283.4 ± 1.0	0.281 ± 0.001	− 69.3 ± 0.75
F30	40	13,750	5	285.6 ± 5.9	0.287 ± 0.001	− 69.2 ± 0.57
F31	40	13,750	5	297.9 ± 8.5	0.293 ± 0.000	− 68.9 ± 0.14
F32	40	13,750	5	274.4 ± 4.6	0.296 ± 0.002	− 70.6 ± 0.05
F33	40	13,750	5	261.8 ± 2.8	0.304 ± 0.001	− 70.9 ± 0.11

**Table 4:** Sequential model sum of squares and model summary statistics for PS, PDI and ZP responses.

	Source	Sum of squares	DF	Mean square	F value	*p* value	Remarks
Particle size	Mean vs. Total	3.110E+06	1	3.110E+06			
	Linear vs. Mean	8620.64	3	2873.55	2.37	0.0828	
	2FI vs. Linear	9529.14	3	3176.38	2.94	0.0435	
	Quadratic vs. 2FI	34940.42	3	11646.81	37.88	< 0.0001	Suggested
	Cubic vs. Quadratic	9484.83	3	3161.61	38.49	< 0.0001	Aliased
	Residual	3121.47	38	82.14			
	Total	3.176E+06	51	62264.72			
PDI	Mean vs. Total	48035.41	1	48035.41			
	Linear vs. Mean	544.38	3	181.46	1.55	0.2130	
	2FI vs. Linear	2498.24	3	832.75	12.26	< 0.0001	
	Quadratic vs. 2FI	1344.15	3	448.05	11.16	< 0.0001	Suggested
	Cubic vs. Quadratic	1315.73	3	438.58	50.51	< 0.0001	Aliased
	Residual	329.98	38	8.68			
	Total	54067.89	51	1060.15			
Zeta potential	Mean vs. Total	2.417E+05	1	2.417E+05			
	Linear vs. Mean	146.97	3	48.99	6.59	0.0008	
	2FI vs. Linear	165.33	3	55.11	13.19	< 0.0001	
	Quadratic vs. 2FI	108.23	3	36.08	19.56	< 0.0001	Suggested
	Cubic vs. Quadratic	29.27	3	9.76	8.00	0.0003	Aliased
	Residual	46.36	38	1.22			
	Total	2.422E+05	51	4748.57			

Model summary statistics							
	Source	Std. dev.	*R*²	Adjusted *R*²	Predicted *R*²	PRESS	
Particle size	Linear	34.85	0.1312	0.0758	-0.0195	66978.04	
	2FI	32.87	0.2763	0.1776	0.0466	62634.67	
	Quadratic	17.53	0.8081	0.7660	0.6824	20863.68	Suggested
	Cubic	9.06	0.9525	0.9375	0.9343	4315.50	Aliased
PDI	Linear	10.81	0.0902	0.0322	-0.1258	6791.11	
	2FI	8.24	0.5044	0.4368	0.2626	4448.44	
	Quadratic	6.34	0.7272	0.6673	0.5245	2868.29	Suggested
	Cubic	2.95	0.9453	0.9280	0.8936	641.98	Aliased
Zeta potential	Linear	2.73	0.2962	0.2513	0.1361	428.65	
	2FI	2.04	0.6294	0.5789	0.4651	265.40	
	Quadratic	1.36	0.8476	0.8141	0.7433	127.35	Suggested
	Cubic	1.10	0.9066	0.8771	0.8148	91.87	Aliased

### ANOVA analysis

The results of the ANOVA analysis (Table 5) demonstrate the quadratic model’s relevance and adequacy for describing the relationships between variables for all responses with F values and p values of 19.19 and < 0.0001 for PS, 12.14 and < 0.0001 for PDI, 25.33 and < 0.0001 for ZP respectively. Regression Eqs. (2, 3, and 4) represent the coded models derived using response surface methodology (RSM). The variables A, B, and C are dimensionless coded values of the actual factors used to normalize the data and improve model fitting. Specifically, A corresponds to the drug amount (30–50 mg), B to the homogenization speed (6500–21,000 rpm), and C to the homogenization time (2–8 min). These models are used to assess the influence of each factor on particle size, polydispersity index (PDI), and zeta potential, furthermore, explained by 3D response surface plots (Fig. 2).

#### Effect of independent variables on particle size (Y1)

The prepared ETX-NCs were found to have PS values of 186.5 ± 1.8 nm to 297.9 ± 8.5 nm, Table 3. ANOVA results showed that the amount of ETX had a statistically significant effect on particle size (*p* = 0.0003), with an effect size of η² = 0.072. This means that approximately 7% of the variation in particle size is explained by the drug concentration, indicating a moderate impact compared to other formulation variables. Homogenization time had a significant effect on the particle size of the nanocrystals (*p* = 0.002), accounting for approximately 5% contribution of the variance (η² = 0.050). This indicates a small but potentially meaningful influence compared to other formulation variables. In contrast, homogenization speed showed a much smaller effect size (η² = 0.009), explaining less than 1% of the variation in particle size. This suggests that within the tested range (6500, 13,750, and 21,000 rpm), homogenization speed had a negligible impact on particle size. The polynomial equation for particle size (PS) (Y₁), presented below as Eq. 2, shows an inverse relationship with the amount of ETX, as indicated by the negative coefficient (-13.99 A). This suggests that increasing the drug concentration promotes nucleation, likely due to the greater availability of drug molecules that accelerate the formation of initial crystal nuclei. Instead of producing fewer, larger particles, this enhanced nucleation results in a higher number of smaller crystals. Moreover, the drug molecules may contribute to stabilizing the nanoparticle growth process, leading to the formation of smaller and more uniformly sized particles^47,48^. However, a positive relationship between homogenization time (C) and particle size was observed, with prolonged homogenization time leading to an increase in particle size. This effect can be explained by the aggregation of very fine particles, which results in the formation of larger particles over time^49^.

2 $$\begin{aligned} {\text{Y1}} & =+{\text{28}}0.{\text{66}}-{\text{13}}.{\text{99 A}}+{\text{5}}.0{\text{7 B}}+{\text{11}}.{\text{73 C}}+{\text{14}}.0{\text{6 AB}}+{\text{14}}.{\text{49 AC}} \\ & \quad - {\text{19}}.{\text{66 BC}} - {\text{7}}.{\text{32 A2}} - {\text{47}}.{\text{75 B2}}-{\text{16}}.{\text{6}}0{\text{ C2}} \\ \end{aligned}$$

#### Effect of independent variables on PDI (Y2)

PDI values of all prepared NCs were between 0.252 ± 0.00 and 0.492 ± 0.00 (Table 3), so all formulations provided homogenous dispersion of NCs. Regarding the following Eq. (3), the tested levels of each independent variable have a slight effect on particles dispersion which is indicated by low coefficient values for each independent variable (Eq. 3). However, time of homogenization provides p value of 0.0008 with effect size of 0.087, indicating that approximately 8.7% of the variability in PDI can be attributed to this factor. This reflects a moderate influence within the context of the studied formulation variables.

3 $$\begin{aligned} {\text{Y2}} & =+{\text{32}}.{\text{54}} - 0.{\text{8413A}} - 0.{\text{3238B}} - {\text{4}}.{\text{68C}} - {\text{1}}0.{\text{42AB}} \\ & \quad +{\text{9}}.{\text{35AC}} - {\text{3}}.{\text{49 BC}} - {\text{8}}.{\text{96 A2}} - {\text{ }}0.{\text{5}}0{\text{97 B2}}+{\text{5}}.{\text{53 C2}} \\ \end{aligned}$$

#### Effect of independent variables on ZP (Y3)

Zeta potential provides an indication about stability of prepared NCs. ZP ranged between − 74.10 ± 0.61 and − 63.70 ± 0.80 (Table 3). Based on results from ANOVA, significant effect of ETX amount (*p* < 0.0001) and time of homogenization (*p* = 0.0004) was observed, where the amount of drug showed a large effect (η² = 0.238), accounting for approximately 24% of the variability. In contrast, homogenization time had a smaller impact (η² = 0.056), indicating a limited but potentially meaningful contribution to zeta potential variation. Regarding Eq. 4, a positive relation is observed between ETX amount and surface negativity^50^. These results are in accordance with PS results as increased drug amounts resulted in smaller particles, which have higher surface area-to-volume ratio, allowing the drug molecules and lecithin to align or arrange more effectively on the surface, thereby increasing stability^51^. A decrease in ZP was observed upon prolonging time of homogenization (negative coefficient with C, Eq. 4) which is attributed to agglomeration or coalescence of nanoparticles. As particles aggregate, the effective surface area decreases, reducing the exposure of charged groups responsible for maintaining a high zeta potential. This diminishes the repulsive forces between particles, leading to a drop in zeta potential.

4 $$\begin{aligned} {\text{Y3}} & = - {\text{69}}.{\text{83}}+{\text{2}}.{\text{22 A}}+0.{\text{2333 B}} - {\text{1}}.0{\text{8 C}}+{\text{1}}.{\text{61AB}}+{\text{2}}.{\text{17 AC}} \\ & \quad +{\text{2}}.{\text{54 BC}} - 0{\text{l}}.{\text{4625 A2}} - 0.{\text{3292 B2}}+{\text{2}}.{\text{9}}0{\text{ C2}} \\ \end{aligned}$$

**Table 5:** ANOVA analysis.

Parameters	Y1: Particle size			Y2: PDI			Y3: Zeta potential		
	F value	P value	Interpretation	F value	P value	Interpretation	F value	P value	Interpretation
Model	19.19	<0.0001	Significant	12.14	<0.0001	Significant	25.33	<0.0001	Significant
A		0.0003	Significant		0.5190	Not significant		0.0001	Significant
B		0.1638	Not significant		0.8035	Not significant		0.4049	Not significant
C		0.0021	Significant		0.0008	Significant		0.0004	Significant
AB		0.0082	Significant		0.0001	Significant		0.0002	Significant
AC		0.0066	Significant		0.0001	Significant		0.0001	Significant
BC		0.0004	Significant		0.0636	Not significant		0.0001	Significant
A2		0.1456	Not significant		0.0001	Significant		0.2331	Not significant
B2		0.0001	Significant		0.7764	Not significant		0.3940	Not significant
C2		0.0017	Significant		0.0034	Significant		0.0001	Significant

## Characterizations of prepared ETX-NCs

### Drug content

It was found that the drug content in optimized NCs (F5) was 101.89 ± 0.76% w/v. According to these findings, NCs offered the drug’s maximum loading capacity.

### Electron microscopy analysis

**Fig. 3:**
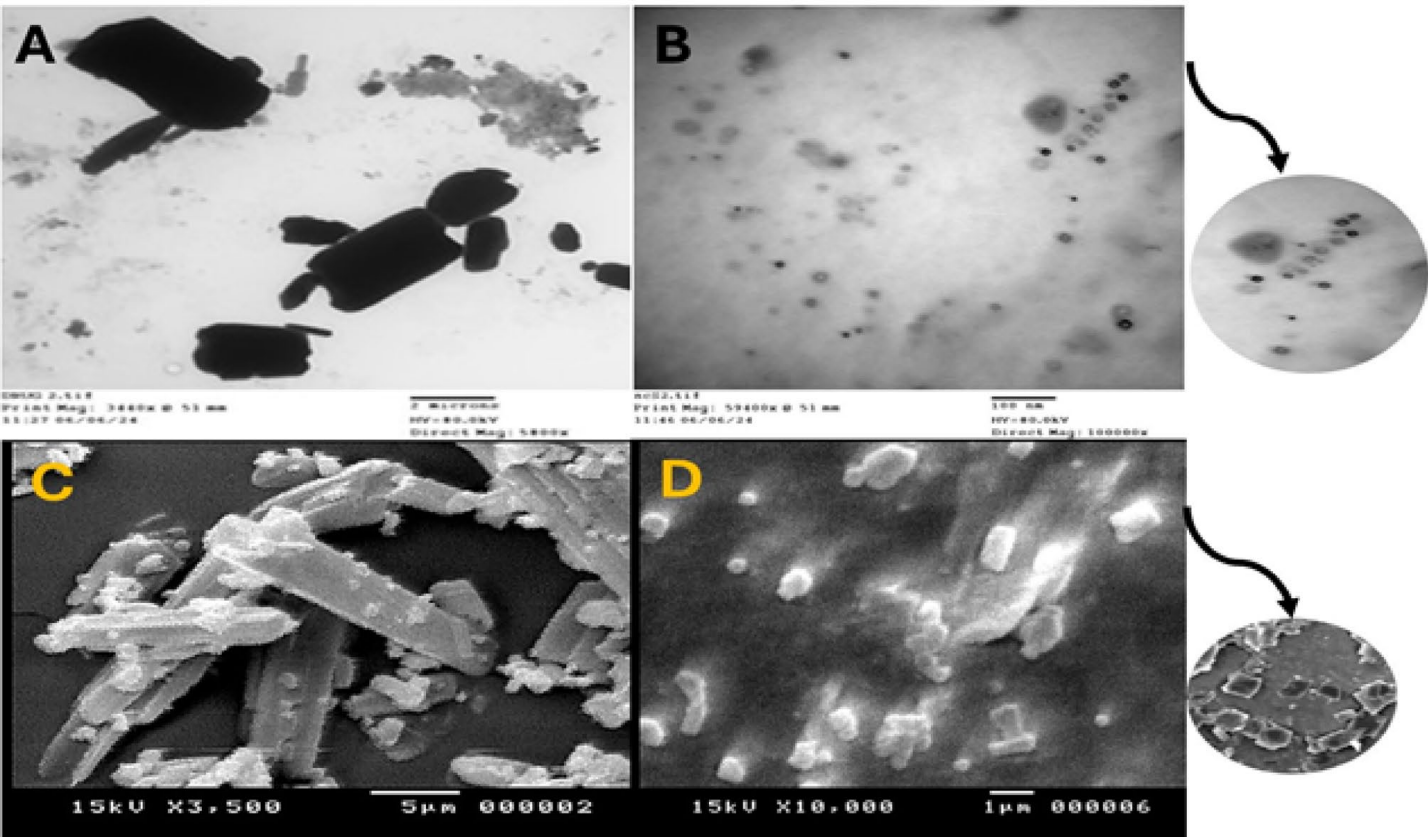
Morphological analysis of pure ETX and ETX-NCs. **A** Transmission electron microscopy (TEM) image of pure ETX. **B** TEM image of ETX-NCs. **C** Scanning electron microscopy (SEM) image of pure ETX. **D** SEM image of ETX-NCs.

Figure 3 illustrates the morphology of pure ETX and ETX-NCs as observed through transmission electron microscopy (TEM) and scanning electron microscopy (SEM). The ETX-NCs exhibited nanoscale particle size with a uniform distribution and a distinct cubic crystalline structure. In contrast, the unprocessed (raw) ETX displayed larger, microscale particles with smooth surfaces. These observations were further supported by SEM analysis.

### Fourier infrared spectroscopy (FT-IR)

**Fig. 4:**
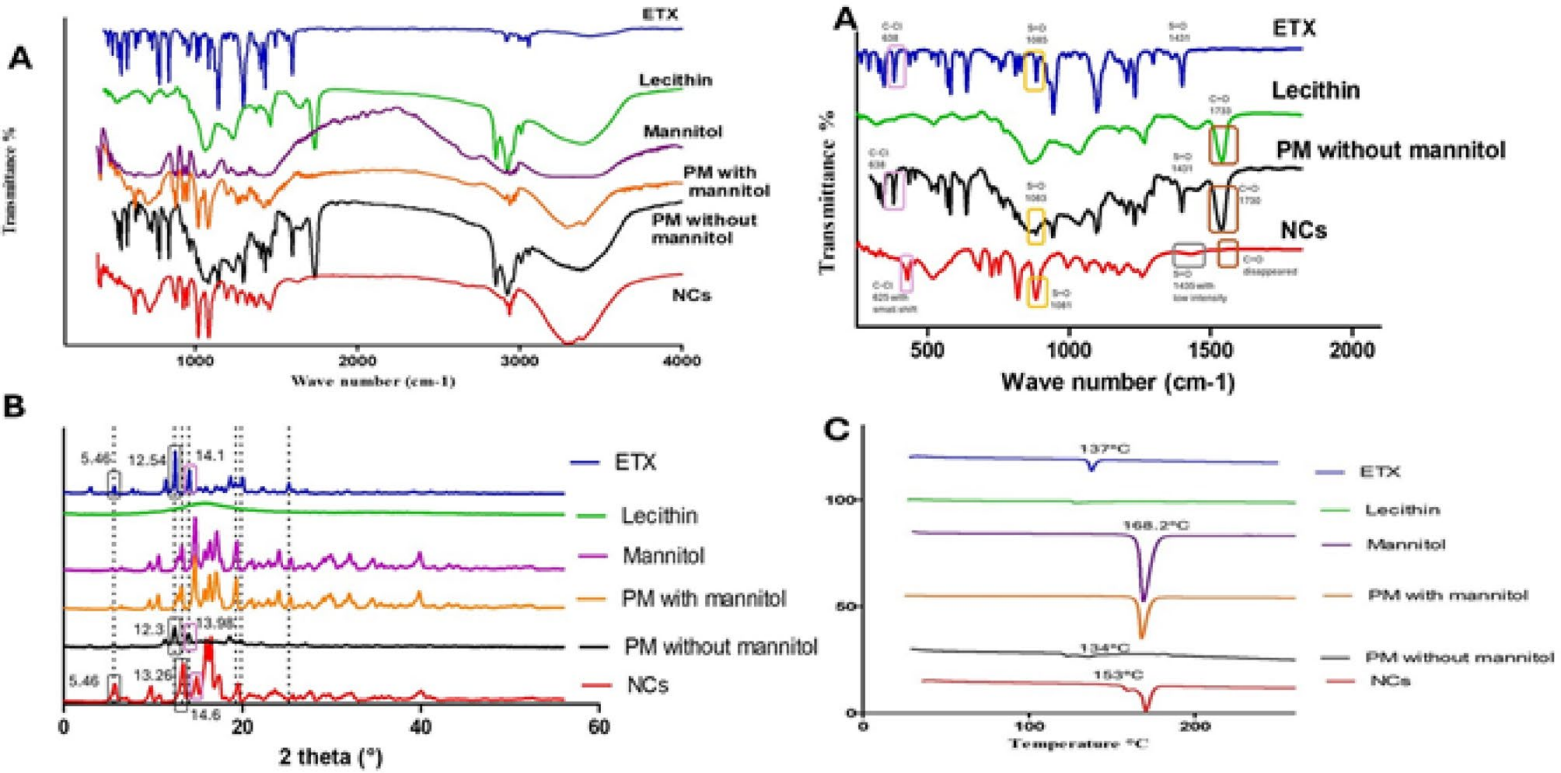
FT-IR spectra (**A**), PXRD (**B**) and DSC thermograms (**C**) of pure ETX, lecithin, mannitol, physical mixture with mannitol, physical mixture without mannitol and ETX-NCs formula.

FTIR spectroscopic analysis was performed to investigate the possible interaction and compatibility of ETX and other excipients. The results (Fig. 4, A) show the characteristic peaks of ETX as 1596.9 cm^−1^ (C = N stretching vibration); 1431 cm^−1^, 1300 cm^−1^, 1141.8 cm^−1^, and 1085.8 cm^−1^ (S = O stretching vibrations); and 840.9 cm^−1^, 775.3 cm^−1^, and 638 cm^−1^ (C-Cl stretching vibrations) respectively^52^. The characteristic peaks in the FT-IR spectrum of lecithin include the alkane bands that correspond to the symmetric CH_2_, antisymmetric CH_2_, antisymmetric CH_3_ stretching, and CH_2_ scissoring vibrational modes at 2854, 2928, 2956, and 1462 cm^− 1^, respectively; the carbonyl stretching vibration at 1736 cm^− 1^, and the highly overlapped PO^2^ − and P–O–C infrared active vibrations in the region between 1200 and 970 cm^− 1^^53^. These peaks were retained in the physical mixture devoid of mannitol, demonstrating ETX’s compatibility with other components. Considering the NCs’ FT-IR spectrum, it was found that some ETX distinctive peaks (1433.3 cm^−1^ and 1085.4 cm^−1^) were retained with lower intensity while others underwent small shifts or vanished. Additionally, a distinctive lecithin peak at 1739 cm^−1^ disappeared along with minor shifts of others, indicating molecular interactions between ETX and lecithin in NCs that are consistent with lecithin’s stabilizing impact. Mannitol’s particular peaks were the hydroxyl function (3100–3500 cm^− 1^, very strong broad band), along with additional absorptions at 2935 (C–H), 1081, and 1019 cm^− 1^ (C–O)^54^. Due to the high concentration of mannitol (5%w/v), the IR spectra of the physical mixture containing mannitol mostly displayed the characteristic peaks of mannitol with the elimination of ETX and lecithin peaks. As a result, the sample was diluted.

### Differential scanning colorimetry (DSC)

The DSC thermograms of ETX, lecithin, and their physical mixture (Fig. 4, C) provided complementary information regarding the thermal properties and crystallinity of ETX in the presence of lecithin. Pure ETX displayed a sharp endothermic peak at 137 °C, corresponding to its melting point, which reflects its crystalline nature^55^. Lecithin showed a smaller, broader peak at 126 °C, indicating its relatively lower melting point and more amorphous structure. When combined in the physical mixture (ETX and lecithin), the endothermic peaks were still observed. In the thermogram of NCs, a shift in endothermic peak of ETX to a higher melting point was observed which can be related to the lecithin-induced stabilization that resulted in improved molecular packing in the crystal lattice, this stronger crystalline lattice arrangement requires more energy to break, hence a higher melting temperature^56–59^. Mannitol thermogram exhibited an endothermic melting peak at 168.2 °C which still existed in NCs thermogram. Physical mixture with mannitol (ETX, lecithin and mannitol) showed a single endothermic peak typical to that of mannitol with absence of drug and lecithin peaks which related to high dilution of the sample examined with mannitol.

### Powder X-ray diffraction

The X-ray diffraction (XRD) analysis was performed for pure etoricoxib (ETX), lecithin, mannitol, the physical mixture with mannitol, the physical mixture without mannitol, and the NCs formulation (Fig. 4, B). The XRD pattern of ETX displayed distinct peaks at diffraction angles of 5.46°, 11.46°, 12.54°, 14.1°, 15.96°, 17.1°, 18.66°, 19.98°, 20.1°, and 25.2°^60^. These characteristics to the NCs formulation, some of ETX peaks subjected to slight shifts which related to presence of mannitol, indicating its dilution. Considering XRD pattern of physical mixture with mannitol, disappearance of ETX peaks was observed or it mainly showed the characteristic peaks of mannitol only^61^.

### Saturation solubility

**Fig. 5:**
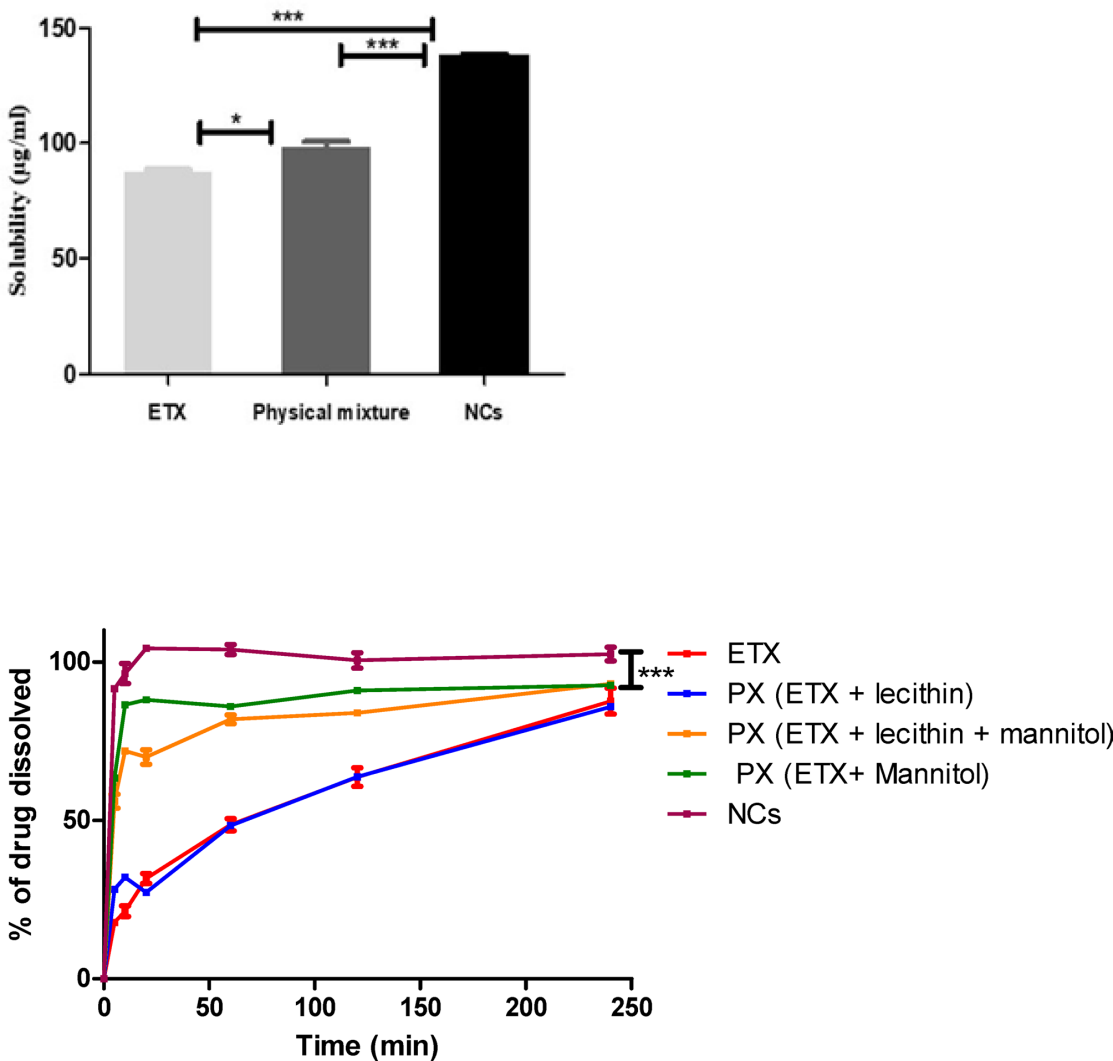
**a** Solubility study of ETX, physical mixture (ETX + lecithin + mannitol) and selected NCs formula (F5), one way ANOVA was used for statistical analysis; *** (*P* < 0.001) and * (*p* < 0.05). **b** In vitro dissolution profiles of ETX, physical mixture (ETX + lecithin), physical mixture (ETX + lecithin + mannitol), physical mixture (ETX + mannitol) and selected NCs formula (F5), ANOVA was used for statistical analysis; *** (*P* < 0.001). significant increase in dissolution by NCs compared to others after 5 min.

Saturation solubility of ETX, Physical mixture (ETX, lecithin and mannitol) and the selected NCs formula was conducted in phosphate buffer (pH 7.4) to investigate the effect of nanocrystal form on ETX solubility. Based on the presented results (Fig. 5a), a significant increase in the saturation solubility of ETX after its inclusion as NCs (137.75 ± 1.34 µg/ml, p value < 0.001) compared to that of pure ETX and the physical mixture (87.70 ± 1.41 µg/ml and 97.95 ± 3.88 µg/ml). A slight enhancement in ETX solubility was achieved in its physical mixture form (p value < 0.05) which can be explained by the presence of mannitol in the mixture acting as a solubilizing agent for ETX through improvement its wettability^62^. In this study, the pH was maintained constant (7.4) to avoid any change in pH as the solubility of ETX, a pH dependent drug, may be affected by any change in the pH during the experiment.

### In-vitro drug dissolution

Pure ETX, physical mixture (ETX + lecithin), physical mixture (ETX + lecithin + mannitol), physical mixture (ETX + mannitol) and selected NCs formula were subjected to dissolution study. The results (Fig. 5b) illustrated the significant role of NCs formation in enhancement of ETX dissolution, where 91.49 ± 0.01% (p value < 0.001) of drug was dissolved after the first 5 min with complete drug dissolution after 10 min. This result can be attributed to the nano-size of ETX-NCs which increase the surface area exposed for dissolution, also, presence of mannitol in the lyophilized NCs has a synergistic effect on ETX dissolution^63^. To investigate and confirm the effect of mannitol, different physical mixtures were studied and the results showed that, for pure ETX, physical mixture (ETX + lecithin), physical mixture (ETX + lecithin + mannitol) and physical mixture (ETX + mannitol), the % of drug dissolved were 17.87 ± 0.01%, 28.29 ± 0.03%, 56.05 ± 3.07% and 63.37 ± 1.23%, respectively after 5 min. These results approved the effect of mannitol in increasing wettability and solubilization of ETX, so improving its dissolution profile was achieved compared to pure form^32,62^.

## Conclusion

Etoricoxib nanocrystals were prepared using acid-base precipitation method, with some modifications. Preparing conditions of 0.1% w/v of lecithin and 30 mg ETX formulated at pH 5.5 under homogenization speed 21000 rpm for 5 min were selected as the most suitable conditions for stabilization of nanocrystals with minimum particle size and PDI based on results of Box–Behnken design. The compatibility of ETX with other ingredients and its crystallinity were determined via FTIR, DSC and X-ray diffraction techniques. ETX-NCs achieved enhancing in etoricoxib saturation solubility, thereby, dissolution rate improved. Consequently, nanocrystals are a promising approach for enhancing etoricoxib properties. The pharmacokinetics of ETX-NCs and its impact on osteoarthritis are being investigated as a part of the ongoing research.

## Data Availability

The datasets analyzed during the current study are available from the corresponding author on reasonable request.
